# The spectrum of Alzheimer’s disease

**DOI:** 10.1101/2025.11.17.25340406

**Published:** 2025-11-19

**Authors:** J.S. Novotny, M. Čarná, I. Lyburn, T. Kuruvilla, G.B. Stokin

**Affiliations:** 1 Institute of Molecular and Translational Medicine, Faculty of Medicine and Dentistry, Palacky University Olomouc, Olomouc, Czech Republic; 2 Cobalt Medical Charity, Cheltenham, UK; 3 Cranfield University, Bedford, UK; 4 Gloucestershire Health & Care NHS Foundation Trust, Gloucester, UK; 5 University of Gloucestershire, Cheltenham, UK; 6 Gloucestershire Hospitals NHS Foundation Trust, Gloucester, UK

**Keywords:** Alzheimer’s disease, mild cognitive impairment, dementia, diagnostics, hippocampus, inferior parietal lobule, choroid plexus, cognitive scores, cerebrospinal fluid Ab, tau and phospho-tau

## Abstract

While hippocampal (H) and inferior parietal lobule (IPL) atrophy are used routinely in Alzheimer’s disease (AD) diagnostics, the role of enlarged choroid plexus (ChP) remains unclear. We here examined the AD Neuroimaging Initiative (ADNI) cohort (N=872) to investigate the contribution of enlarged ChP in predicting AD using MRI volumetry. Analyses revealed that no individual volumetric brain changes, nor their combination, can predict AD. Among AD patients, only ~ 19%, 12% and 5% exhibited changes in H, ChP or IPL volumes, while 45% showed no volumetric brain changes at all, not even longitudinally. Amyloid-b peptides, therefore, contribute to brain atrophy and neuronal loss at best only in a subset of amyloid PET-CT positive AD patients. These findings suggest that despite shared amyloidopathy, the observed brain volumetric phenotypes, together with their corresponding cognitive and CSF biomarker profiles, represent unique entities within the AD spectrum much like the synucleinopathies, tauopathies and TDP43-proteinopathies.

## Introduction

Alzheimer’s disease (AD) is characterized clinically by cognitive decline and pathologically by neurodegeneration in addition to its neuropathological hallmark lesions, namely senile plaques and neurofibrillary tangles^1^. Neurodegeneration has been extensively reported to correspond clinically to brain atrophy^2^. Aberrant accumulation of extracellular amyloid-b peptides (Ab) has been demonstrated to underlie the formation of senile plaques, while hyperphosphorylation of microtubule-associated tau protein gives rise to neurofibrillary tangles. In contrast to neurofibrillary tangles, which can be observed in several brain disorders, senile plaques are found predominantly in AD brains. This has led to the amyloid cascade hypothesis as the most widespread explanation for development of AD and driven the generation of recently introduced Ab targeting therapies^3–6^. Ab can now be measured in the cerebrospinal fluid and imaged directly in the brains^7,8^.

Reduced size of medial temporal lobes, including the hippocampi (H), the inferior parietal lobules (IPL) and other brain structures^9,10^, has long been used as an imaging biomarker of neurodegeneration in AD diagnostics^11,12^. In contrast to the reduced size of these structures, several research groups have recently reported not a decrease but an increase in the size of the choroid plexus (ChP) in AD^13–16^. Several studies have also reported that the size of ChP increases already with normal aging and even more so in AD, where it undergoes significant remodelling and inversely correlates with cognitive decline^14,17^. Despite all these recent findings, the possible contribution of increased ChP size to AD diagnostics has not yet been addressed. Here, we investigated the role of ChP in AD diagnostics. Unexpectedly, we found that no volumetric brain changes can predict AD, that amyloid peptides cause brain atrophy and neuronal loss at best only in a subset of AD patients, and lastly, describe different clinical phenotypes within the AD spectrum.

## Results

**Extended Data Figure 1.**
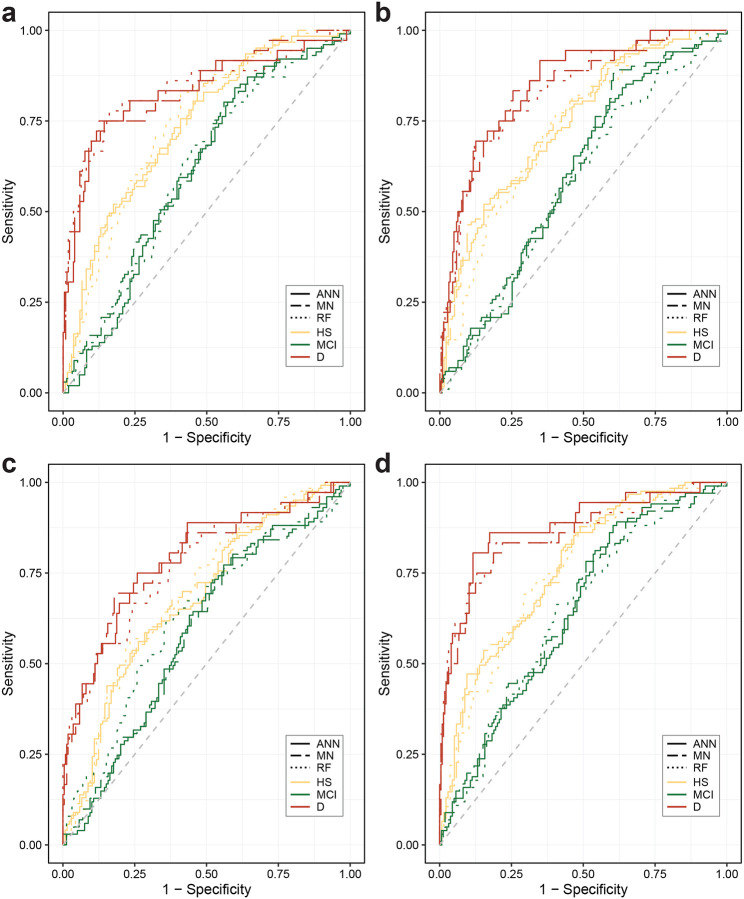
Multiclass prediction of diagnostic AD status based on brain region volumes. ROC curves of multiclass prediction of diagnostic status (HS, MCI, dementia) based on combined presence of (**a**) H and IPL atrophy, (**b**) H atrophy and enlarged ChP, (**c**) IPL atrophy and enlarged ChP, and (**d**) H and IPL atrophy and enlarged ChP using three different prediction methods. Abbreviations: HS: healthy subjects, MCI: AD-related mild cognitive impairment, D: Alzheimer’s dementia, ANN: artificial neural network, MN: multinomial logistic regression, RF: Random Forest.

**Extended Data Figure 2.**
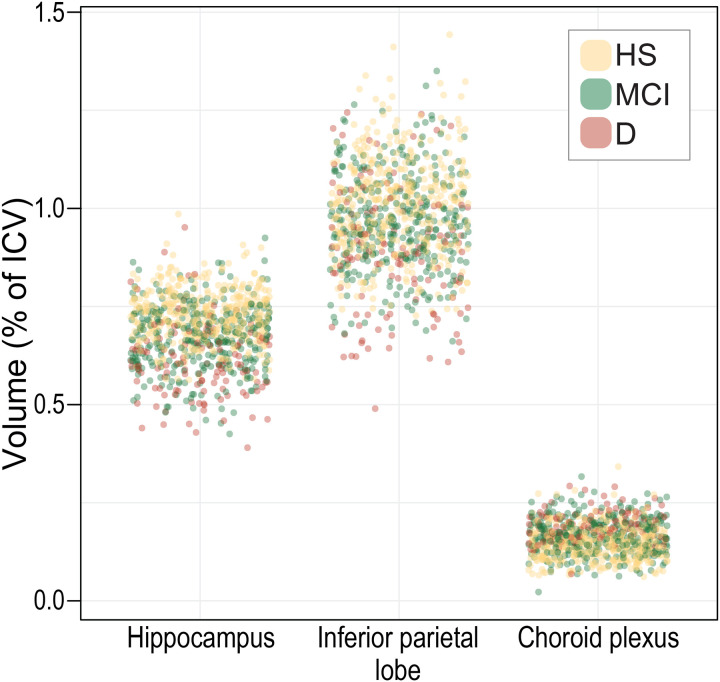
Distribution of brain region volumes in individual cases based on diagnostic status within age groups. Abbreviations: HS: healthy subjects, MCI: AD-related mild cognitive impairment, D: Alzheimer’s dementia.

**Extended Data Figure 3.**
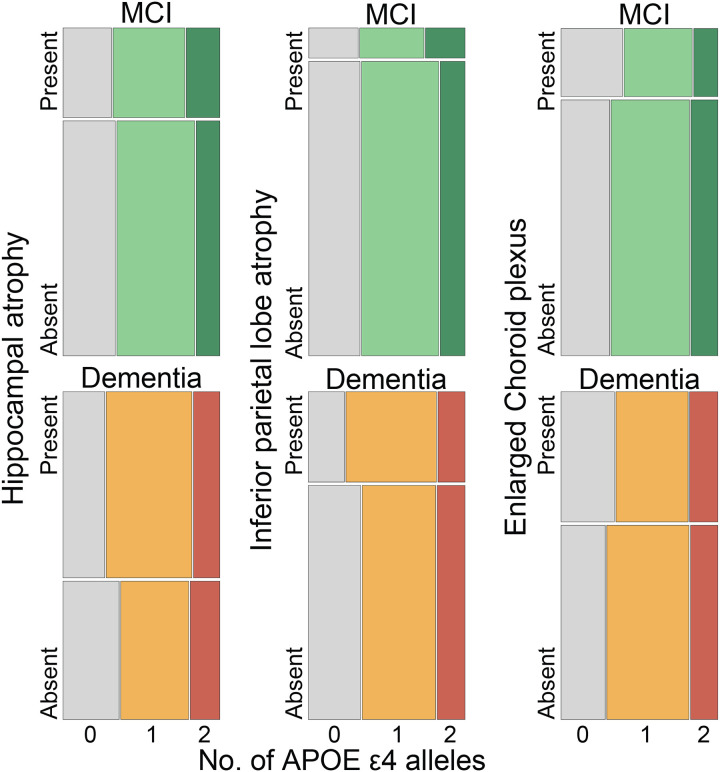
Prevalence of the number of ApoE ɛ4 alleles depending on the brain volumetric changes in MCI and dementia cases.

**Figure 1.**
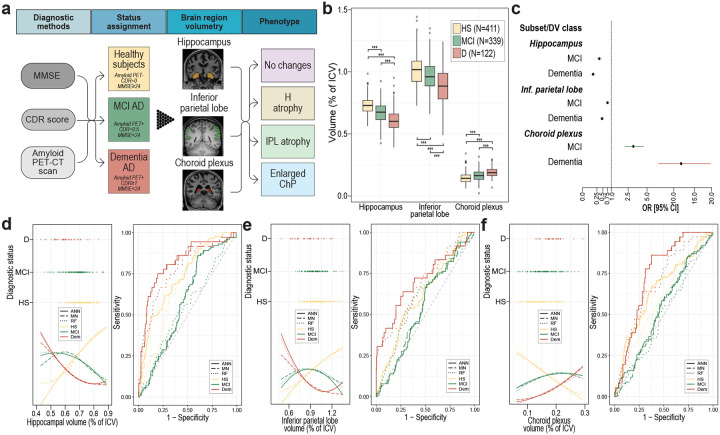
The association between brain volumetric changes and AD status. (**a**) The pipeline depicts the process of establishing diagnostic status and obtaining brain volumetric change-based phenotypes. (**b**) Mean brain volumes in individual diagnostic AD statuses (HS, MCI, D). (**c**) Multinomial logistic regression odds ratios of belonging to AD-related diagnostic groups (MCI and D) depending on H, IPL or ChP volumes (bound to a change in volume of 0.1). (**d-f**) Multiclass prediction of diagnostic status (classes: HS, MCI, D) based on (**d**) H, (**e**) IPL and (**f**) ChP volumes using multinomial logistic regression. Left point-line plot shows the distribution of H, IPL or ChP volumes across diagnostic groups (points), with lines indicating the predicted probabilities of diagnosis (depending on H, IPL or ChP volume) for individual diagnostic statuses using three different prediction methods. Right plot shows ROC curves for individual diagnostic statuses. Abbreviations: HS: healthy subjects, MCI: AD-related mild cognitive impairment, D: AD dementia, DV: dependent variable, ANN: artificial neural network, MN: multinomial logistic regression, RF: Random Forest. ***P<0.001

To examine the potential role of the ChP in AD diagnostics, we measured mean volumes of the H, IPL and ChP in healthy subjects and amyloid PET-CT positive AD patients with either MCI or dementia. As expected, H and IPL volumes progressively decreased from healthy subject to MCI and dementia, whereas ChP mean volumes progressively increased across the groups (Figure 1b, Supplementary Table S1). We next calculated odds ratios, using multinomial logistic regression. All three volumetric brain measures showed a significant association with AD diagnostic status. This indicated that changes in H, IPL, and ChP volumes are associated with increased odds of MCI and dementia (Figure 1c, Supplementary Table S2). Despite these positive associations, however, none of the applied predictive models - artificial neural networks, multinomial logistic regression, or Random Forest - was able to reliably predict diagnostic category, HS, MCI, or dementia, based on H, IPL, or ChP volumes, either individually or when combined (Figure 1d, e and f, Extended Data Figure 1, Supplementary Table S3). This was partly driven by inherent variability in volumetric brain measures within each diagnostic group (Extended Data Figure 2). Finally, we found that the presence of ApoE e4 alleles had no effect on the examined brain volumes (Extended Data Figure 3, Supplementary Table S4).

**Extended Data Figure 4.**
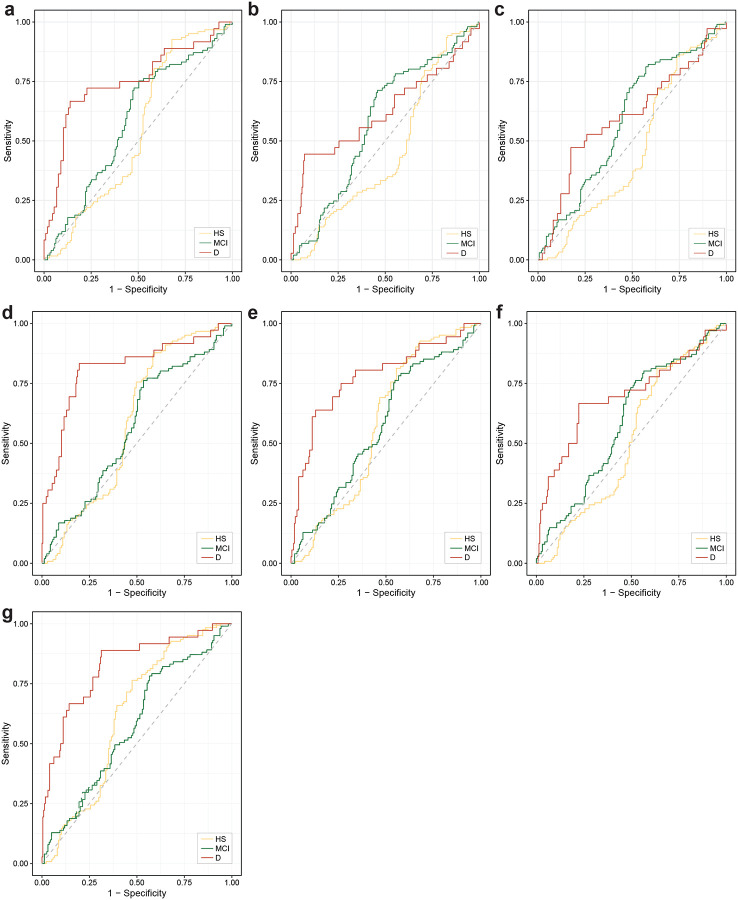
Multiclass prediction of diagnostic status based on individual and combined presence/absence of regional volumetric changes. Predictions are shown based on the presence of (**a**) H atrophy, (**b**) IPL atrophy, (**c**) enlarged ChP, (**d**) H and IPL atrophy, (**e**) H atrophy and enlarged ChP, (**f**) IPL atrophy and enlarged ChP, and (**g**) H and IPL atrophy and enlarged ChP using artificial neural network. Abbreviations: MCI: AD-related mild cognitive impairment, D: Alzheimer’s dementia.

**Figure 2.**
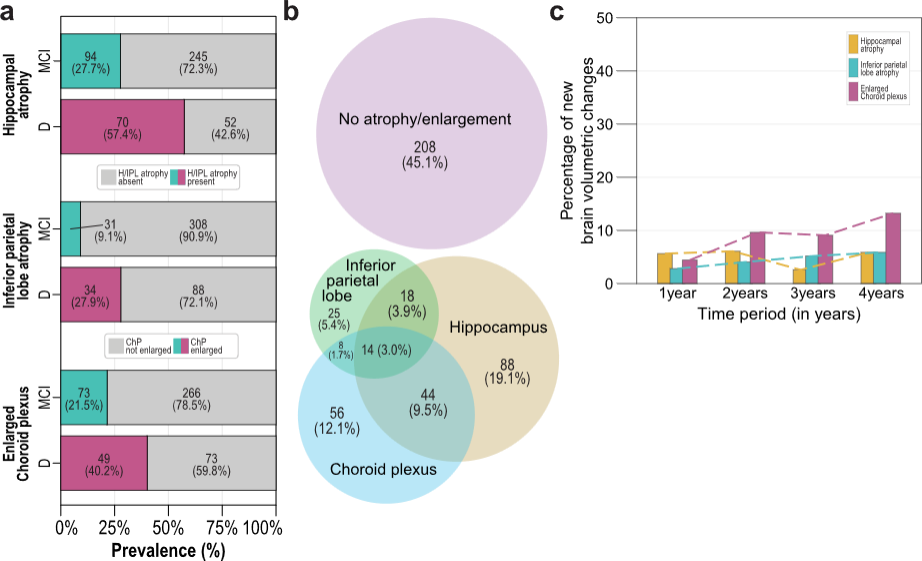
Prevalence of brain volumetric changes in amyloid PET-CT positive MCI and dementia cases. (**a**) Prevalence of H, IPL and ChP volumetric changes in AD MCI and dementia. (**b**) Venn diagram showing the extent of overlap between cases with H, IPL and ChP volumetric changes and the number and perecentage of cases without any volumetric changes within MCI and dementia cases. (**c**) Longitudinal changes in the incidence of H, IPL and ChP volumetric changes. Bars indicate the percentage of new cases that manifested development of H, IPL or ChP volumetric changes in a defined period (in years). The rest of the cases (85–95% on average) in the given period displayed no changes. Abbreviations: HS: healthy subjects, MCI: AD-related mild cognitive impairment, Dem: Alzheimer’s dementia.

We next investigated volumetric brain changes in amyloid PET-CT–positive MCI and dementia patients. In MCI, only 27.7 %, 9.1 % and 21.5 % of patients showed H atrophy, IPL atrophy, or enlarged ChP, respectively. These proportions increased in the dementia group to 57.4 %, 27.9 %, and 40.2 %, respectively (Figure 2a, Supplementary Table S5). Despite the observed brain volumetric changes, almost half of all AD patients exhibited no volumetric brain changes at all. Among those with changes, only 19%, 5 % and 12% showed changes exclusively in H, IPL, or ChP volumes (Figure 2b). Longitudinal analyses revealed that the incidence of new ChP enlargement approximately doubled over the four-year follow-up, whereas the incidence of new H or IPL atrophy remained largely unchanged during the same period (Figure 2c, Supplementary Table S6). Finally, multiclass modelling using artificial neural networks was unable to predict AD status based on the presence of H or ILP atrophy or enlarged ChP (Extended Data Figure 4, Supplementary Table S7).

**Extended Data Figure 5.**
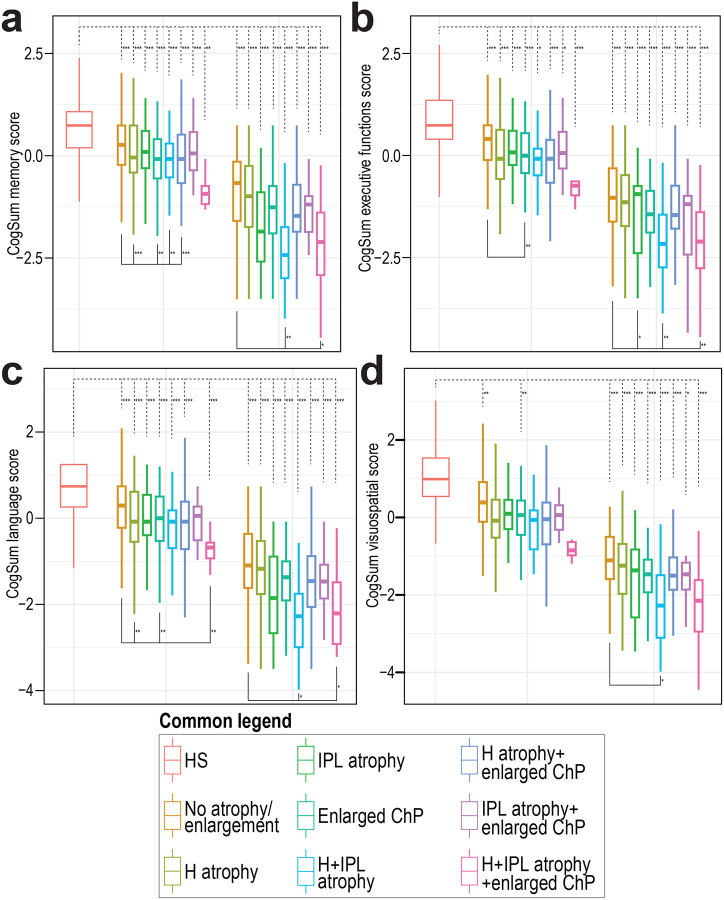
Cognitive performance according to ADNI CogSum scores based on presence of volumetric brain changes. Boxplots show (**a**) ADNI’s CogSum memory score, (**b**) ADNI’s CogSum executive functions score, (**c**) ADNI’s CogSum language score, and (**d**) ADNI’s CogSum visuospatial score in individual groups according to the presence of H and IPL atrophy and enlarged ChP and their combinations. Values are plotted separately for the healthy subjects, MCI and dementia subsets. Upper dashed-line brackets indicate significant differences relative to the HS group, while lower solid-line brackets indicate significant differences relative to the No volumetric brain change group. Abbreviations: HS: healthy subjects, H: Hippocampus, IPL: inferior parietal lobe, ChP: Choroid plexus. *P<0.05, **P<0.01, ***P<0.001

**Figure 3.**
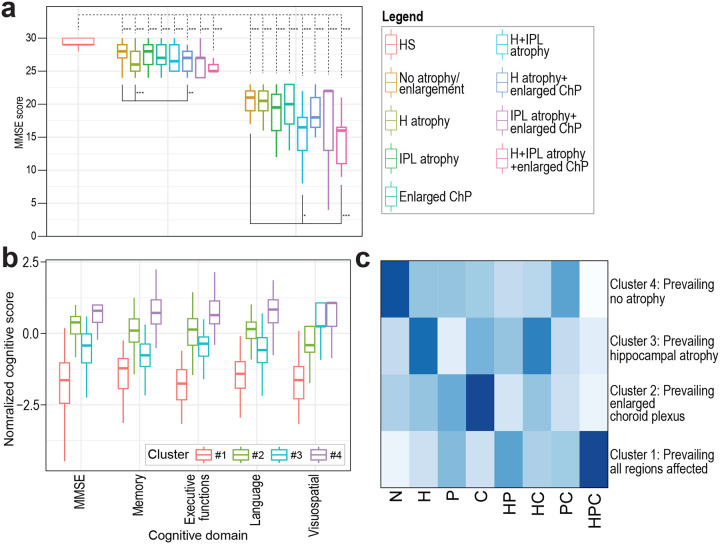
Cognitive performance based on brain volumetric changes. (**a**) MMSE scores in individual groups according to the presence of H, IPL or ChP volumetric changes. Values are plotted separately for the healthy subjects and MCI and dementia subsets. Upper dashed-line brackets indicate significant differences relative to HS, while lower solid-line brackets indicate significant differences relative to the group without volumetric changes (no brain volumetric changes). (**b**) Boxplots of scaled MMSE and ADNI CogSum cognitive scores in individual identified clusters. (**c**) Proportion of amyloid PET-CT positive cases with brain volumetric changes in individual clusters based on hierarchical cluster analysis of scaled MMSE and ADNI CogSum cognitive scores. Colour scale represents row percentages of groups with brain volumetric changes within a cluster (i.e. proportion of groups in cluster). Abbreviations: HS: healthy subjects, N: No volumetric brain change, H: Hippocampus, IPL: Inferior parietal lobe, ChP/C: Choroid plexus, HP: Hippocampal and inferior parietal lobe atrophy, HC: Hippocampal atrophy and enlarged choroid plexus, PC: inferior parietal lobe atrophy and enlarged choroid plexus, HPC: Hippocampal and inferior parietal lobe atrophy and enlarged choroid plexus. *P<0.05, **P<0.01, ***P<0.001

Mini-Mental State Examination (MMSE) scores showed that, independently of brain volumetry, all amyloid PET-CT-positive MCI and dementia patients exhibited worse cognitive performance than healthy subjects (Figure 3a, Supplementary Table S8). Rare patients who exhibited H and IPL atrophy together with enlarged ChP showed the most profound cognitive deterioration. A similar pattern was observed across individual cognitive domains, as reflected by the ADNI CogSum scores (Extended Data Figure 5, Supplementary Table S9).

To explore cognition further, we looked for similarities in cognitive profiles and identified four unique clusters (Figure 3b, Supplementary Table S10). Cluster 1 showed severe cognitive deterioration across all domains. Cluster 2 demonstrated cognitive decline with impairments in memory, executive functions, and language, but not so much in visuo-spatial abilities. Cluster 3 exhibited milder cognitive decline with impairments in all cognitive subdomains. Cluster 4 displayed the least severe cognitive deficits. Intriguingly, these cognitive profiles corresponded closely to different patterns of volumetric brain changes. Cluster 1 represented predominantly patients with simultaneous H and IPL atrophy as well as enlarged ChP. Cluster 2 was enriched in patients with isolated H atrophy, cluster 3 in patients with enlarged ChP, while cluster 4 in those without any detectable volumetric changes (Figure 3c, Supplementary Table S11)

**Extended Data Figure 6.**
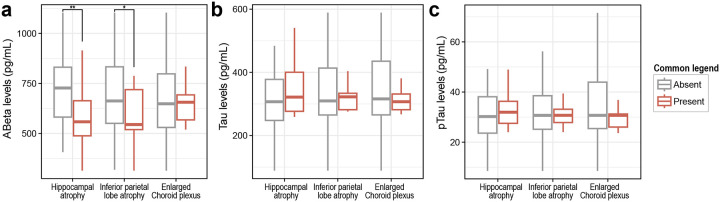
Levels of CSF biomarkers depending on the brain volumetric changes in amyloid PET-CT positive MCI and dementia cases. Boxplots show differences in (**a**) Ab levels, (**b**) tau levels, and (**c**) phospho-tau levels based on presence or absence of individual volumetric brain changes. *P<0.05, **P<0.01

Finally, we examined whether cerebrospinal fluid (CSF) AD biomarkers were associated with volumetric brain changes. AD patients with H or IPL atrophy, but not those with enlarged ChP, exhibited significantly reduced CSF Ab levels compared with patients without atrophies. In contrast, CSF tau and phospho-tau levels showed no changes between AD patients with or without brain atrophy or enlarged ChP (Extended Data Figure 6, Supplementary Table S12).

## Discussion

Although all mean volumetric brain changes were associated with AD, none of them, individually nor in combination, could predict AD. This is partly due to inherent variability in volumetric brain measures within experimental groups, but more importantly due the fact that most AD cases showed no volumetric brain changes at all, and only a minority exhibited specific patterns of brain atrophy and/or enlarged ChP. In contrast to earlier diagnostic frameworks^11,12^, our results demonstrate that volumetric brain changes are not optimal diagnostic biomarkers for AD. Volumetric brain changes can, therefore, be considered at best supportive, but not defining AD.

All MCI and dementia AD patients in our cohort were amyloid PET-CT positive, but almost half of them showed no detectable brain atrophy, while others exhibited diverse combinations of volumetric changes in select brain regions. These findings indicate that Ab can cause brain atrophy and neuronal loss at best only in a subset of AD patients, thus challenging the amyloid cascade hypothesis^3,4^. This divergence also raises the possibility that Ab targeting therapies^5,6^ may be most effective in AD patients with H and IPL atrophy, as these individuals showed the most significant reductions in CSF Ab levels and may therefore carry the highest brain amyloid load^18^. Although unlikely, it is plausible to entertain the possibility that different volumetric phenotypes represent a continuum of changes in time, where one first exhibits no volumetric brain changes, then ChP begins to enlarge to compensate for Ab pathology, and last develops H and/or IPL atrophy.

Our findings reveal the presence of multiple clinical phenotypes within the AD spectrum. For example, AD patients with H atrophy exhibited severe cognitive decline affecting memory, executive functions and language, but not so much visuospatial abilities, and displayed the most significant reduction in CSF Ab levels. In contrast, AD patient with enlarged ChP exhibited milder cognitive decline across all cognitive subdomains and showed no CSF Ab changes compared with AD cases without volumetric changes. These distinct clinical, brain volumetric and biochemical profiles are difficult to reconcile under a single pathogenic mechanism. Past neuropathological studies of AD also support the existence of multiple AD phenotypes^19^. Some of these phenotypes may correspond to known clinical subtypes, for example IPL atrophy may represent an earlier or milder form of posterior cortical atrophy^20^. Equally, enlarged ChP may well represent those cases that possess the capacity of augmented Ab clearance. Other may result from coexisting pathologies, such as aberrant accumulation ofTDP-43 or α-synuclein^21,22^. Alternatively, these phenotypes may represent distinct disease entities within the broader Alzheimer’s spectrum, unified by amyloid deposition but differing fundamentally in the underlying mechanisms of neurodegeneration. In this framework, AD would encompass multiple amyloidopathies, each arising from defects in different steps of Aβ production or clearance within specific brain cell populations. These disorders would share aberrant Aβ accumulation but diverge in their cognitive profiles, volumetric signatures, CSF biomarker patterns, and underlying pathogenic cascades - paralleling how synucleinopathies, tauopathies, and TDP-43 proteinopathies represent families of disorders centred around a shared protein anomaly.

## Methods

### Source of the data

The data were obtained from the AD Neuroimaging Initiative (ADNI) database (www.adni.loni.usc.edu). For up-to-date information, see www.adni-info.org.

### Sample

All participants who completed cognitive screening (Mini Mental State Examination (MMSE), AD Neuroimaging Initiative (ADNI) CogSum and Clinical Dementia Rating (CDR) scale) and underwent head MRI and amyloid PET-CT imaging were included in this study. This led to a final sample of 872 participants with an average age of 71.77±7.04 years, of whom 444 (50.9%) were women. The sample was then stratified into three groups according to AD diagnosis (Figure 1a). Participants with an amyloid-negative PET-CT scan, CDR score of 0, and MMSE score >=24 were classified as healthy subjects (abbrev. HS, N=411, mean age=70.2±6.6), participants with an amyloid-positive PET-CT scan, MMSE score >=24 and CDR score of 0.5 were classified as MCI AD cases (abbrev. MCI, N=339, mean age=72.8±6.8) and finally, participants with an amyloid-positive PET-CT scan, MMSE score <24 and CDR score of 1 or more were classified as dementia AD cases (abbrev. D, N=122, mean age=74.2±7.9). A subset of participants had their Apolipoprotein ε4 status established, as well as cerebrospinal fluid Ab, tau, and phosphor-tau biomarker measurements. Data on the number of APOE4 alleles was available for 855 (98%% of the entire sample) cases. Data on the levels of individual CSF biomarkers was available for 159 (18.2% of the entire sample) cases.

### MRI images

The data were based on ADNI MRI images^23^. The study was focused on examining the volumes of three regions of interest: the hippocampus (H), inferior parietal lobe (IPL) and choroid plexus (ChP)(Figure 1a). As a source of volume data, we used pre-processed MRI volume data produced by UCSF and available in the ADNI repository. Specifically, we used the cross-sectional dataset UCSFFSX7, including participants from ADNI1, GO, 2, 3, and 4, and the longitudinal dataset UCSFFSL51. T1-weighted MRI images were processed using FreeSurfer v.7.x for cross-sectional data and v.5.1 for longitudinal data. A detailed description of data processing has been reported previously^24,25^. For participants for whom data from multiple MRI images at different timepoints were available, we selected representative MRI image data. Specifically, we calculated the average of the normalized volumes of regions of interest (with inverted normalized values for the choroid plexus) for each of the participant’s MRI images and selected the one with the mean average volume value. Volumes of regions of interest were calculated as sum of volumes of given region counted by UCSF and normalized against intracranial volume to control for the effect of brain shrinkage with age on the volume of regions of interest^26,27^.

### Definitions of volumetric brain changes

Cases with H and IPL atrophy were defined as those whose volume was below the 5th percentile of the normative population^28^. Since no specific thresholds for determining enlarged ChP have been defined yet, we derived our own working threshold based on the threshold for the hippocampus and by examining the distribution of ChP volumes. We considered cases with enlarged ChP to be those whose volumes were at or above the 91st percentile of the normative population. The normative population in this study consisted of healthy subjects.

### Amyloid PET-CT scans, APOE- ε4 and CSF biomarkers processing

Cerebral Aβ deposition was visualized using previously validated pipeline across different PET tracers (^11^C-Pittsburgh Compound B (PiB),^18^F-florbetapir (FBP), ^18^F-florbetaben (FBB), and ^18^F-NAV4694 (NAV))^29^. PET imaging was performed within 2 weeks before or after the baseline clinical assessments, as described previously^30^. Amyloid positivity was defined by UC Berkeley^29,31^.

The *APOE- ε4* carrier status was identified from DNA extracted by Cogenics from a 3 ml aliquot of EDTA blood extracted from participants during their screening visit^32^.

For CSF biomarkers, lumbar puncture was performed as described in the ADNI procedures manual (http://www.adni-info.org/). CSF samples were frozen on dry ice within 1 hour after collection and shipped overnight on dry ice to the ADNI Biomarker Core laboratory at the University of Pennsylvania Medical Center. Aliquots (0.5 mL) were prepared and stored in barcode-labeled polypropylene vials at −80°C. Never-before-thawed aliquots of CSF samples were used in this study. CSF samples were measured using the Elecsys β-amyloid(1–42) and the Elecsys phospho-tau (181P) and Elecsys total-tau immunoassays on a cobas e 601 analyzer (software version 05.02) at the Biomarker Research Laboratory, University of Pennsylvania, USA (ADNI), according to the preliminary kit manufacturer’s instructions and as described in previous studies^33^.

### Statistical analysis

No imputation of missing values was performed; only complete cases selected previously were included in the study. Differences in brain region volumes (BRV) between diagnostic statuses were analysed using ANCOVA testing with controls for sex, age, and education. The odds of belonging to a diagnostic status based on BRV were tested using multinomial logistic regression. The predictability of diagnostic status based on BRV values was verified using artificial neural networks, random forests, and multinomial logistic regression, while predictions based on the volumetric brain changes and their combinations were verified using artificial neural networks.

Differences in the prevalence of volumetric brain changes and in the number of APOE4 alleles between MCI and dementia were analysed using the chi-squared test and Cramer’s V as the effect size. The presence of significant trends in the longitudinal incidence of volumetric brain changes was analysed using the Mann-Kendall Trend test and Sen’s slope for trend magnitude. Differences in cognitive scores between groups based on the combination of volumetric brain changes were analysed using ANOVA with Dunnett’s post hoc test against healthy subjects and those without volumetric brain changes. Differences in the levels of CSF biomarkers depending on the volumetric brain changes were analysed using a t-test. Finally, individual AD patients were classified into clusters based on their cognitive performance using hierarchical cluster analysis.

All statistical analyses were performed as two-tailed and all the P<0.05 were considered statistically significant. The Benjamini-Hochberg P-values correction was applied in every case of multiple pairwise comparisons. Data analysis and visualizations were performed in RStudio (v.2022.07.2 with R environment v.4.2.1).

## Extended Data

## Supplementary Material

Supplement 1

## Data Availability

ADNI data are available from the online repository after approved registration (https://adni.loni.usc.edu/). No new original data were generated in this study.
